# Medical professionalism in the formal curriculum: 5^th^ year medical students’ experiences

**DOI:** 10.1186/s12909-014-0259-0

**Published:** 2014-11-30

**Authors:** Amelia J Stockley, Karen Forbes

**Affiliations:** Department of Palliative Medicine, University of Bristol, Horfield Road, Bristol, BS2 8ED UK

**Keywords:** Professionalism, Curriculum development, Faculty development, Formal curriculum, Ticking-boxes

## Abstract

**Background:**

The standards and outcomes outlined in the General Medical Council’s publication ‘Tomorrow’s Doctors’ include proposals that medical professionalism be included in undergraduate curricula. Learning the values and attitudes necessary to become a ‘doctor as a professional’ has traditionally been left largely to the informal and hidden curricula. There remains no consensus or confirmed evidence upon which to base best practice for teaching in this area. In 2010, as part of a revision of the fifth year curriculum the University of Bristol Medical School introduced tutorials which focused on students’ achievement of the learning objectives in ‘Tomorrow’s Doctors *Outcomes 3: the doctor as a professional’.* This study sought to explore the students’ experiences of these tutorials in order to develop the evidence base further.

**Methods:**

Sixteen medical students participated in three focus-group interviews exploring their experiences of medical professionalism tutorials. A course evaluation questionnaire to all fifth year students also provided data. Data were analysed using the principles of Interpretative Phenomenological Analysis.

**Results:**

Four main themes were identified: students’ aversion to ‘ticking-boxes’, lack of engagement by the students, lack of engagement by the tutors and students’ views on how medical professionalism should be taught.

**Conclusions:**

A curriculum innovation which placed the achievement of medical professionalism in the formal curriculum was not unanimously embraced by students or faculty. Further consideration of the students’ aversion to ‘ticking-boxes’ is warranted. With continued demand for increased accountability and transparency in medical education, detailed check-lists of specific learning objectives will continue to feature as a means by which medical schools and learners demonstrate attainment. Students’ experiences and acceptance of these check-lists deserves attention in order to inform teaching and learning in this area. Learner and faculty ‘buy in’ are imperative to the success of curriculum change and vital if the students are to attain the intended learning objectives. Effective faculty development and student induction programmes could be employed to facilitate engagement by both parties.

## Background

The standards outlined in the General Medical Council’s (GMC’s) ‘Tomorrow’s Doctors’ published in 1993 and its revisions include recommendations that professionalism be included in undergraduate medical education in the United Kingdom (UK) [1]. Whilst diversity of curriculum approach in UK medical schools is encouraged, all schools must ensure that the learning outcomes specified in Tomorrow’s Doctors are attained by their students by graduation [2]. In 2010 the University of Bristol revised the fifth year medical curriculum to incorporate the outcomes for graduates in the updated Tomorrow’s Doctors of 2009 [3]. All ‘Tomorrow’s Doctors’ outcomes were examined and themed by one of the authors (KF) into 12 areas and the students’ understanding and current achievement of these outcomes was to be discussed within twelve weekly tutorials. The new curriculum included seven weekly small-group tutorials of between 10 and 15 students focusing on the learning objectives proposed in *Outcomes 3: the doctor as a professional*, specifically items 20 a, c, d, e, f, g and 23 c, d, e, f, g, h, i, j, placing medical professionalism in the formal fifth year curriculum.

Despite an increase in theory and research regarding medical professionalism in the last decade, there is ‘no clear consensus or evidence base to inform best practice, teaching and evaluation in this area’ [4]. We know that student satisfaction is important for learning; whilst a positive reaction is no guarantee of learning, a negative reaction almost certainly reduces its possibility [5]. Insights into the students’ experiences of learning medical professionalism in a formal curriculum are vital, therefore, to informing and enhancing curriculum development and teaching practice in this area. The aim of this qualitative study was to explore students’ views and experiences of specific medical professionalism tutorials in the formal curriculum within their revised fifth year undergraduate medical course.

## Methods

### Participants

The study focused on fifth year medical students all of whom participated in the new curriculum of 2010/2011. The experiences of the entire year were identified as pertinent to the study’s aims and objectives.

### Data collection

After obtaining ethical approval for the study from the University Faculty of Medicine and Dentistry Committee for Ethics, all fifth year medical students were invited via email to complete an on-line evaluation of the Preparing for Professional Practice course within which the professionalism tutorials sat. The students completed an anonymous questionnaire, responses being voluntary and confidential. All students were invited to take part in semi-structured focus-group interviews as they completed their evaluation questionnaire.

The course evaluation questionnaire included free-text response questions in which a number of students wrote about the tutorials. These on-line survey responses were accessible through the students’ virtual learning environment after completion of the survey and gave an insight into students’ individual experiences.

Focus-group interviews were carried out to explore feedback from the students regarding their shared experiences of the group tutorials. The focus-groups involved semi-structured, face-to-face group interviews performed by a single researcher and an observer. Written consent obtained from each of the students prior to interviews included consent to audio-recording and the use of anonymous quotes in publications, posters and the study report. Interviews lasted between 60 and 90 minutes and were based on a topic-guide derived from the literature. The audio-files of the interviews were transcribed verbatim by a transcriber experienced in qualitative research.

### Data analysis

Data were analysed using Interpretative Phenomenological Analysis (IPA). IPA is a qualitative research approach which seeks to describe people’s perceptions of their experience and explore how they derive meaning from them [6]. Interpretation takes place at two levels; through the recollection of an experience the participant will try to interpret and make sense of the experience. The researcher then interprets the accounts to discover common meanings. This method was judged more relevant for this study than grounded theory, the main alternative method for this sort of qualitative research, because of its focus on experience rather than on the generation of theory.

The audio-files, transcripts and questionnaire responses were analysed using the six-step structured approach outlined by Smith, Flowers and Larkin [6]*.* Analysis of the transcripts and of the questionnaire responses relevant to the tutorials was performed contemporaneously. The process involved the recognition of patterns and connections across the data and the establishment of themes and subthemes that were pertinent and applicable to the whole data set. A second researcher (KF) independently read the transcripts and concurred with the identified themes and sub-themes.

## Results

220 students were invited to complete the evaluation questionnaire and asked whether they would participate in focus-group interviews. 155 students completed the questionnaire (response rate 70.5%). Fifty six students commented on the tutorials within free-text responses. Nineteen students declaring an interest in participating in the focus-group interviews were emailed an information sheet. Through email dialogue three focus-group interviews were arranged for the sixteen students able to attend comprising five, four and seven students respectively. Of the students able to attend the focus-group interviews eleven were female and five male, with fifteen being of Caucasian and one of Asian ethnicity.

The quotations taken from the focus-group transcripts are in normal font below whilst those from the on-line survey are presented in italics.

### Students’ aversion to ticking-boxes

A number of the students disliked the notion that the tutorials were based on a checklist which tallied with the ‘Tomorrow’s Doctors’ objectives. They were unable to articulate why this was although one student suggested that it was ‘disheartening’ or ‘a shame’. Others used language like ‘shove’ and ‘whack’ to describe the manner in which the curriculum changes were made and delivered. Some students, whilst acknowledging that the GMC’s learning objectives needed to be covered in their curriculum, felt that the checklists were instituted in order that the university could be ‘seen to be doing something’.

My tutor, by contrast, was just tick boxing the whole time. She made it really obvious that she was like, “You have to do this, this, this, this, this, and we’re just going to tick the boxes as we go.” Group 2.

But I get the sense that there were some tutors who were so prescriptive that they just followed this list, tick, tick, tick. Group 3.

And it’s almost like the medical school thought, “Oh we haven’t done that, we’d better whack it in at the end of the fifth year.” Group 1.

So it was just really like, “Oh tick all this, let’s just shove it through.” Group 1.

It was really disheartening. It was like…Yeah but having a tutor tell me that…we just had to tick boxes. And it’s like oh that’s such a shame, even though we know it’s true. Group 2.

I certainly got the sense, in quite a lot of this course, that our teaching package was in some ways built to be shown to be seen to be doing something. Group 3.

### Lack of engagement by students

#### Relevance

Many of the students were unable to see the relevance of the tutorials. The content of the tutorials was referred to using words such as ‘random’ and ‘bizarre’.

*Fewer core tutorials…many are not relevant and interfere with being able to complete shadowing and ward work. Student 16.*

I mean…bizarre, to be honest…I just thought, I don’t know, I don’t know where they are coming from. Because yeah Tomorrow’s Doctors is fine, but just taking tutorial headings and then saying, “That’s what you’re going to do for an hour, clinical governance,” I mean what about it? Group 3.

*Tutors covered seemingly random topics, they appeared to have had little guidance on how to make the sessions worthwhile. Student 20.*

Other students were able to see the relevance to their future practice but were disappointed by what was delivered.

The topic of the tutorials that we had were not…and I mean that’s not saying they’re not relevant to our career at all, but they were just massive overkill, like talking…for three hours, it’s quite obvious. Group 1.

And a lot of the things, like clinical governance, it’s good to know about, but they will have to cover that in our F1…so it kind of just felt like it’s almost relevant but not quite, so just leave it alone. Group 3.

### We know this already

Several of the students seemed puzzled that medical professionalism needed to be addressed explicitly in the formal curriculum believing that they had covered it elsewhere in the curriculum.

I think that some of the topics they were covering were just things that we have been in contact with for six years. Group 1.

And one of the tutorials was meant to be about culture, “Present a case for discussion of a patient with a cultural angle to their care.” And if that hasn’t been throughout the course up to now, I don’t know what has been really. Group 1.

It’s just a bit weird, because…people are working with professionalism from day one. Group 3.

*Tutorials on clinical governance, professional behaviour, infection control* etc. *This should already be known to us by final year! Student 84.*

*While I understand that these are important topics, I feel that they do not need to be taught as they have been covered throughout the course in different ways and are well understood by final year students. Student 140.*

### Patronising

A number of the students felt being asked to address the learning outcomes in Tomorrow’s Doctors: *‘the doctor as a professional’* was patronising, with some of the issues discussed in the tutorials being ‘obvious’ or ‘tedious’.

….And to kind of go over it step by step with learning objectives was a bit patronising, and well I think it was just slightly unnecessary. Group 1.

*Re-think some of the PPP tutorials* e.g. *infection control, equality and diversity… they were hugely patronising and not very useful. Student 148.*

*The group tutorials can be very patronising and unnecessary. Student 120.*

### Lack of engagement by the tutors

#### Variable interpretation of the learning objectives

The students acknowledged tutors’ variable approaches to the tutorials. Some students were pleased that their tutor had the ‘imagination’ to address the subject in a way which was relevant to the students. Others felt a more student-centred approach rather than doggedly tackling each learning objective (‘shoving it through’) might have been preferable or less ‘dry’.

Different tutors interpret it very differently. And I think I was lucky to have a tutor who took one look at the form and went, “I’ll just put this to one side and then I’ll teach you what I think you need to know.” And I think I was lucky, because he took the initiative, he saw what Bristol had provided for him and thought, “Hmm OK,” and then he used his own imagination. And there was a bit of a lack of that, I found…throughout the course, where people took what Bristol had given them and then applied it without really thinking it through very well. And, you know, our tutor was really good. Group 3.

And again you will get some people (tutors) who are facilitating and are just, “I don’t care about this,” and some that go, “They (the tutorials) are brilliant.” Group 1.

I think it is worthwhile having those explicit times for that exploration. But I think maybe it just needs to be made clear, clearer to the facilitator that it’s not just about forcing it through for three hours, but about a space to be able to explore. Group 1.

And I think, you know you were saying that the tutorials were aimed at Tomorrow’s Doctors, I don’t think our tutor had that focus. Group 2.

*Change the themed tutorials as these were exceptionally dry topics and tutors had to be inventive to keep us interested. Student 19.*

### Not taking it seriously

The students experienced a variety of approaches to delivering the learning objectives through the tutorials, some more favourable than others; it seemed some of the tutors did not engage with the learning outcomes in Tomorrow’s Doctors: *Outcomes 3*.

Because a lot of the tutors themselves seem to think that this whole thing is an absolute joke. Group 1.

I think as well you have to get the tutors to sort of want to give the tutorial. Because I just felt in some of ours that kind of if they thought it was a joke, what on earth were we meant to think? Group 1.

*(Get rid) of tutorials such as infection control that even the tutors don’t think is a good use of anyone's time. Student 94.*

One of the students identified the irony in being taught about medical professionalism by a tutor who was not demonstrating exemplary professional behaviour.

Things like a consultant saying, “Oh yeah, so just be professional,” and then being really rude and just awful about patients and about colleagues that he’d worked with and things. And you sit there and you think, you know, we’re having a tutorial on professionalism, and you’re not taking it seriously. Group 1.

### Students’ views on how medical professionalism should be taught

#### Can it be taught in the formal curriculum?

The students questioned whether professionalism could be addressed through the formal curriculum.

And can that be done through didactic teaching? I don’t think you could have such a thing as an hour or two session where everyone comes in and then leaves a little bit more professional. I just…I don’t think it’s a working model. Group 3.

### It should remain in the informal or hidden curricula

A number of students felt that learning professionalism should remain opportunistic or centred around experiences on the wards, that is, it should occur in the hidden and informal curricula.

I think you pick up attitudes, kind of professional attitudes on the wards and things really. So it’s almost like what’s ethically right to do, and like you should put the patient first, and you should always look after yourself. And I guess you just pick it up on the wards, I think, in general, and being around people with similar attitudes. Group 3.

Guidelines, they’re not awful, I mean actually they’ve got a really great bunch of ideas. But the idea that you have to tick that and you have to verify everything actually happened definitely, it’s kind of just…it should be embedded everywhere and you shouldn’t have to have a check list. Group 1.

One student pertinently pointed out that learning about professionalism should not occur solely in the final year of medical school and another suggested that medical professionalism should be a vertical theme in the curriculum.

I think it should be done way earlier, not at the beginning of this term. I mean if we’ve got to this stage of the final term of med school, and we’re hopeless at professionalism…that’s not realistic. It is a continual process. Group 3.

(*Get rid of) the compulsory tutorials, as these were generally so dry despite being taught as well as possible by excellent teachers. It would be next to impossible to get something out of them. They could be integrated as a vertical theme. Student 112.*

### Limitations/reflexivity

This study has a number of limitations. First, the researcher’s role as a member of the teaching faculty at the university might have had an impact on the students’ willingness to share their experiences. However, it was clearly stated on the information sheet that the research was not evaluating the participants or their academic performance and the importance of confidentiality was emphasised in the introduction to each focus-group. Similarly, sharing experiences in a group setting with peers might also have influenced the students’ responses. Reassuringly, the free-text responses from the course evaluation provided triangulation of the findings from the focus-groups, in that the same codes and themes were derived from the questionnaire data. This indicates that the potentially threatening face-to-face element of the interview process did not appear to influence the students’ reports of their experiences significantly. Secondly, the students motivated to attend the focus-group interviews or indeed to complete the free text responses of the course evaluation might have had particularly strong opinions about their experiences of their tutorials leading to selection bias. Furthermore, as the course evaluation was anonymised it was not possible to establish whether the views of those students attending the focus-group interviews were also captured in the free-text responses thereby effectively increasing their representation. Thirdly, students were responding to the approach taken by the University of Bristol when it placed medical professionalism in the formal curriculum so responses may not be generalizable to other medical schools. Finally, the small cohort of focus-group participants and free-text responders (taking into account the possible over-representation of some students’ opinions if they provided data through both methodologies) means that only trends and directions can be commented upon, providing ideas upon which to base future research. The themes cannot with certainty be generalized to the remainder of the 5^th^ year medical students who experienced the curriculum change.

## Discussion

Many of the students were disconcerted by the use of the Tomorrow’s Doctors learning objectives linked to individual tutorials and referred to this disapprovingly as ‘tick-boxing’. This might be considered surprising as clearly defined learning objectives could serve to focus students and staff allowing for clear demonstration of accomplishment [7]. However, perhaps the students’ attitudes suggest that tutors who ‘ticked-boxes’ lacked the ‘imagination’ to allow them to tailor more student-centred tutorials to the perceived unmet needs of the students. In an essay published in Academic Medicine in 2007, a doctor who had just given birth is subjected to a post-partum ‘check-list’ interview by a medical student. Papin observes that the check-list allowed the student not to make a personal connection with her and muses on how avoiding this experience needs to be translated into medical education [8]. Perhaps explicit ‘tick-boxing’ in the tutorials removes this personal connection between the students and their tutor and with the learning experience, leading them to feel disheartened. Alternatively it might be that students continue to seek comfort in traditional models of medical apprenticeship in which wisdom and experience are conveyed opportunistically and not through the use of tutorials and check lists. There is very little in the literature on this phenomenon and further research could be conducted here. Specifically, a greater understanding of the students’ disregard for the use of check lists by the tutors in addressing the learning objectives might ensure that faculty can avoid situations that dishearten students.

A significant number of students were unable to see the relevance of the tutorials. They were irritated when the core tutorials took them away from what they perceived to be more useful or relevant learning on the wards. Perhaps the inability to see the relevance of medical professionalism here indicates that the students are unaware that it is a GMC requirement that all graduates have knowledge and skills in this area. Using the model of Johari’s window, it may be that students do not know what they do not know [9] or, indeed, what they need to know. The finding that students do not engage with [10] or ‘buy in’ [11] to teaching on medical professionalism is reflected in the literature. In a study comprising interviews with 56 medical students in the United States of America in 2004/2005 Baernstein et al. reported that ‘people see that professionalism is coming up on the schedule and skip class’ [11]. They suggest that the promotion of ‘learner buy-in’ is essential to engaging students in a topic such as medical professionalism that they may perceive as abstract or extraneous to the scientific curriculum [11]. Detailed induction processes might improve student engagement by providing clarity, contextualising relevance as to what to expect from learning experiences, allowing students to focus on the meaning of medical professionalism, how it pertains to them both as students and also to their future careers, and how medical professionalism as a subject area fits into their wider curriculum. It is acknowledged, also, that assessments or examinations are powerful incentives for students to learn and therefore engage in a subject area. However, there is no consensus as to reliable and valid methods of evaluation regarding medical professionalism [12] and research in this area needs to continue.

Several students described negative experiences of being taught medical professionalism using words such as ‘patronising’ or ‘obvious’. These negative experiences are mirrored in the literature. In Baernstein et al’s study participants used other unfavourable descriptors including ‘insulting’, ‘common sense’ or ‘ambiguous’ [11]. In a study exploring the experiences of faculty in teaching professional attitudes to undergraduates, Stephenson et al. also note students’ antipathy to the subject area: ‘I think it runs off their backs. The ones who are ready to hear those messages and probably are already aware of them, hear them again. The ones to whom it doesn’t make sense, or they think they are being patronised, just say “well this is all common sense” and they get quite angry about it’ [10]. The accusation that some of the learning objectives in this area of the curriculum are simply ‘common sense’ and accordingly ‘patronising’ in their inclusion suggests that the students are perhaps unaware of what informs curriculum design: that medical schools are required to cover the learning outcomes defined by the GMC and demonstrating attainment of these learning objectives is also a vital part of curriculum design. To ensure student engagement and to facilitate learning in this field, steps need to be taken early on to ‘contract’ or engage the students in their learning, perhaps with explanations about how their curriculum has been derived, informed, quality assured and monitored. In this way it may be possible to avoid the elements of teaching that evoke such negative reactions.

The students’ experiences of the core tutorials were tutor dependent. 220 students were timetabled for the weekly tutorials over several sites so there was evidently room for variation despite tutor notes. Some tutors used their ‘imagination’, providing what was perceived to be more relevant teaching. Whether the teaching was more relevant to the intended learning objectives is not clear but it seems plausible that teaching contextualised with perceived relevance for the student is likely to be more meaningful and more readily received. That some tutors are reported to have found the learning objectives ‘a joke’ is worrying but might indicate a lack of understanding about the intentions of the curriculum change and poor faculty development or induction. Steinert et al. suggest that many faculty members themselves are unable to articulate the very professional attributes that they are expected to teach, often believing that, because they are professional, teaching professionalism should be ‘intuitive’ [13]. We are told that teachers can be apathetic towards new and innovative approaches to medical education, instead espousing the teaching and learning of ‘scientific knowledge and the mastery of traditional clinical skills’ [1].

Unfortunately, one student described a situation in which she witnessed a tutor behaving in an unprofessional manner. Modelling of poor attitudes and inappropriate behaviour by teachers and staff can have a deleterious impact on a learner’s development of professionalism [10,14] and it is vital that faculty serve as effective role models at all times [13]. Indeed, Baernstein et al. stress the importance of holding the entire faculty, i.e. all potential role models, to the highest professional standards in order to truly promote professionalism in trainees at medical school [11].

Some students questioned whether medical professionalism could be taught at all in the formal curriculum with one student describing how it was not possible to leave a tutorial after one hour ‘a little bit more professional’. This was clearly never the intention and such over-simplification of the concept reflects a lack of awareness of the meaning of medical professionalism, how it relates to students’ professional body’s guidelines and therefore to the medical curriculum. Attention to student induction may have been helpful here.

A few students felt that medical professionalism should remain in the hidden or informal curricula with no place in the formal curriculum whilst others suggested that lectures or ‘condensed’ tutorials could have a role. The formal curriculum has been identified in some studies as an important adjunct in teaching professionalism, students have found that lectures for example could be ‘inspirational’ or ‘helpful in elucidating observed professional behaviours’ [11]. In Bryden et al’s study of faculty members’ experiences of teaching professionalism participants suggested that the formal curriculum provided the correct forum to discuss ‘institutional and legislative codes’, for example [4]. A combined approach would seem prudent, employing the formal, hidden and informal curricula, to include role-modelling and experiential learning [11].

One student suggested that small group work is important for ‘exploration’ and indeed some authors suggest that students should be provided with opportunities to debrief experiences of negative role modelling with appropriate faculty members in order to ‘mitigate against potentially deleterious effects’ [10,11]. Monrouxe et al. concur, suggesting that the more active or ‘sense-making’ opportunities students have within the formal curriculum ‘the less likely it is that students’ understandings of professionalism will be negatively influenced by the hidden curriculum’ [15].

The observations that the fifth year is ‘too late’ to start teaching medical professionalism and that medical professionalism should be a vertical or spiral theme are prudent. Rabow found that medical students believe that professional skills are not simply procedural skills but ‘proficiencies that develop and are refined with experience’ [16]. Furthermore, Hilton and Slotnick assert that the essential attribute of a mature professional is practical wisdom or ‘phronesis’, i.e. ‘knowing which rules to break and how far to break them to accommodate the reality at hand’ [17]. They describe such insight and judgement as being derived only from experience in managing paradox, complexity and uncertainty. There is thus agreement that becoming the ‘doctor as a professional’ is a developmental process. The notion that medical students can begin to develop professionalism early in their medical studies adds weight to the argument that the teaching and learning of professionalism should be ‘embedded everywhere’, running throughout the entire undergraduate curriculum.

## Conclusions

Approaches to medical education have had to evolve in order to reassure the public that the skills and attributes required by doctors in training can be acquired reliably [18,19]. This ongoing drive for accountability and transparency in medical education means detailed curricula with specific learning objectives referencing GMC guidance will continue to provide the framework for teaching and learning in medical schools for the foreseeable future. As a consequence, check lists and ‘ticking-boxes’ will continue to feature. The finding that a significant proportion of the students demonstrated antipathy towards these check-lists within tutorials provides a new perspective on this method of addressing learning objectives explicitly: students’ experiences and acceptance of ‘ticking-boxes’ need further consideration in order to inform practice in this area.

Many students realised that they had come across teaching relevant to professionalism during their course; it may be that such teaching needs only to be made more explicit. Other studies might include addressing faculty development to ensure faculty consensus, attention to ‘buy-in’ and role-modelling to strengthen and standardise the quality of teaching of professionalism. Studies looking at robust induction processes that would engage, motivate or ‘prime’ students as they enter medical school on learning and being taught medical professionalism would strengthen the current knowledge base. Designing the curriculum with medical professionalism as a vertical or spiral theme would mean medical professionalism would be developed throughout medical school and research is needed to see if this would deepen students’ understanding, awareness and insights into medical professionalism and its applicability.
